# Mango (*Mangifera indica* L.) germplasm diversity based on single nucleotide polymorphisms derived from the transcriptome

**DOI:** 10.1186/s12870-015-0663-6

**Published:** 2015-11-14

**Authors:** Amir Sherman, Mor Rubinstein, Ravit Eshed, Miri Benita, Mazal Ish-Shalom, Michal Sharabi-Schwager, Ada Rozen, David Saada, Yuval Cohen, Ron Ophir

**Affiliations:** Department of Fruit Trees Sciences, Institute of Plant Sciences, Agricultural Research Organization, Volcani Center, Rishon Lezion, Israel; The Robert H. Smith Institute of Plant Sciences and Genetics in Agriculture, Faculty of Agriculture, Food and Environment, The Hebrew University of Jerusalem, Rehovot, Israel

**Keywords:** Mango, Genetic diversity, Transcriptome, SNP, SSR

## Abstract

**Background:**

Germplasm collections are an important source for plant breeding, especially in fruit trees which have a long duration of juvenile period. Thus, efforts have been made to study the diversity of fruit tree collections. Even though mango is an economically important crop, most of the studies on diversity in mango collections have been conducted with a small number of genetic markers.

**Results:**

We describe a *de novo* transcriptome assembly from mango cultivar ‘Keitt’. Variation discovery was performed using Illumina resequencing of ‘Keitt’ and ‘Tommy Atkins’ cultivars identified 332,016 single-nucleotide polymorphisms (SNPs) and 1903 simple-sequence repeats (SSRs). Most of the SSRs (70.1 %) were of trinucleotide with the preponderance of motif (GGA/AAG)n and only 23.5 % were di-nucleotide SSRs with the mostly of (AT/AT)n motif. Further investigation of the diversity in the Israeli mango collection was performed based on a subset of 293 SNPs. Those markers have divided the Israeli mango collection into two major groups: one group included mostly mango accessions from Southeast Asia (Malaysia, Thailand, Indonesia) and India and the other with mainly of Floridian and Israeli mango cultivars. The latter group was more polymorphic (F_S_ = −0.1 on the average) and was more of an admixture than the former group. A slight population differentiation was detected (F_ST_ = 0.03), suggesting that if the mango accessions of the western world apparently was originated from Southeast Asia, as has been previously suggested, the duration of cultivation was not long enough to develop a distinct genetic background.

**Conclusions:**

Whole-transcriptome reconstruction was used to significantly broaden the mango’s genetic variation resources, i.e., SNPs and SSRs. The set of SNP markers described in this study is novel. A subset of SNPs was sampled to explore the Israeli mango collection and most of them were polymorphic in many mango accessions. Therefore, we believe that these SNPs will be valuable as they recapitulate and strengthen the history of mango diversity.

**Electronic supplementary material:**

The online version of this article (doi:10.1186/s12870-015-0663-6) contains supplementary material, which is available to authorized users.

## Background

The origin of *Mangifera indica* L. species which includes all commercial cultivars is still undetermined. The genus *Mangifera* has approximately 70 members which are located mostly on the Malay peninsula, in the Indonesian archipelago, in Thailand and in the Philippines [1, 2]. Some of these species have edible fruit which are locally cultivated. Mango cultivation began a few thousand years ago in India. It first spread from Southeast Asia, only several hundred years ago, with the Portuguese and Spaniards to Africa, Central and South America. In recent years mango has become common in most tropical and subtropical regions. India together with several other countries in Southeast Asia is the main growing and production center for mango. Hundreds of known cultivars has been isolated in the last few hundred years in several mango growing countries, mainly in India, and in the Pacific islands [2]. A secondary mango center flourished in Florida during the late nineteenth century and early twentieth century, and many new Floridian cultivars were promoted [3]. These cultivars are adapted to the taste of the Western consumer by breeding to a red blush coloration, mild taste and mild aroma idoetype. However, there is still some demand for cultivar improvement, and several breeding programs are active in Australia, South Africa, Brazil and Israel [4].

Germplasm collections are important for genotypic and phenotypic analyses, and as a genetic resource in breeding programs. Knowledge of the diversity and the genetic structure of these collections is a fundamental for association studies and controlled breeding [5]. Despite the mango economic importance, the available genetic and genomic resources for mango cannot support modern breeding or the study of the molecular mechanisms underlying mango’s physiology. A limited genetic map with very low resolution has been created for mango [6]. A few studies have attempted to decipher relationship among mango cultivar collections worldwide [7–14]. Twenty five Floridian accessions from the USDA collection were grouped into two clusters based on 28 random amplified polymorphic DNA (RAPD) markers [15]. One cluster was comprised of a group of Floridian accessions that are closely related to the Indian cultivar ‘Mulgoba’ whereas the other cluster contained a group of more distant accessions. A sample from the Floridian groups was also included in a work on the relationship of 22 mango accessions from the Thai mango collection. The variability of the Thai accessions was high and they were not distinguishable from the Floridian accessions, apparently because most of them were seedlings [8]. The Pakistanian collection mostly included Indian-originated mango accessions. Based on RAPD analysis of 44 loci due to high diversity in mango, only the southern Indian accessions could be separated from northern and eastern ones [10]. Two other studies investigated the association between genetic diversity and geographical properties of accessions in India [16, 17]. Those studies weakly separated the northern and eastern accessions from the southern and western ones. A Spanish research group showed that 16 simple-sequence repeats (SSRs) can differentiate the Floridian cultivars from the Indian and the Filipino ones in the 28 accessions of a Spanish collection [18].

Recently, the genetic diversity of mangoes originated from Andhra Pradesh, the major mango breeding area in India, was studied based on 106 polymorphic SSRs. Accessions of the same ideotype (juicy, pickle, table) were more related to each other but did not show any significant differentiation [19]. Further support for the high diversity of mango came from a study of six Colombian cultivar groups showing that the diversity within the six groups is as high as the diversity between them, which indicating very minor population divergence [11]. A broader survey of mango collection, including many geographical locations, was performed in Australia with the caveat of a low number of markers (11 SSRs) [13, 20]. The mangoes were successfully classified into five geographical origins however an attempt to classify the accessions by mono- or polyembryonic phenotype was unsuccessful.

Molecular efforts to create wide genomic and genetic data for mango are in their initial stages. These efforts have included establishment of a leaf transcriptome [21] and fruit transcriptomes at different developmental and ripening stages [22–25]. Next generation sequencing (NGS) technologies are excellent tools for genome-wide marker discovery and exposing genetic variation [26]. *De novo* transcriptome sequencing is one solution for marker discovery, gene expression analysis and exposing genetic variability in organisms with no genomic infrastructure such as olives, Chinese chestnut, carrot and pomegranate [27–31]. Large scale sets of genetic markers can be used to establish genetic maps. These maps can then be utilized for plant breeding and be utilized for anchoring in *de novo* genome assemblies. Moreover, studying the genetic variation of the germplasm collection can give insights into the historical basis of the diversity and can additionally be used for genome wide association studies in order to identify markers linked with important horticultural traits for plant breeding [32].

In the present work we describe our effort to broaden the transcriptome resources for mango by sequencing RNA from various tissues and fruit stages. Using 454-GS FLX Titanium technology we reconstructed a large portion of the ‘Keitt’ mango transcriptome and used it as a reference for aligning resequencing results. Resequencing of the ‘Keitt’ transcriptome itself as well as another mango accession,‘Tommy Atkins’, by Illumina HiSeq 2000 was used to discover a large set of genetic variation. A subset of that variation was utilized in order to explore the Israeli mango collection which comprises cultivars from several world regions.

## Results and discussion

Genic variation is a very useful resource for marker assisted selection (MAS) and association studies. Therefore RNA samples of two mango accessions, ‘Tommy Atkins’ and ‘Keitt’, were obtained from a pool of tissues (young leaves, young inflorescences, young fruit, flesh and peels of mature fruit) as a representative transcriptome (hereafter Pool transcriptome). By pooling we expected to compensate for tissue-specific gene expression. Variation discovery in the transcriptome was performed in two steps. First, *de novo* assembly of the whole transcriptome was performed by 454-GS FLX Titanium sequencing of ‘Keitt’. Second, resequencing of both mango cultivars, ‘Tommy Atkins’ and ‘Keitt’, was aligned to ‘Keitt’ *de novo* assembly contigs to obtain high coverage and therefore high accuracy of allele identification [33].

### Assembly and annotation of the reference transcriptome

**Fig. 1:**
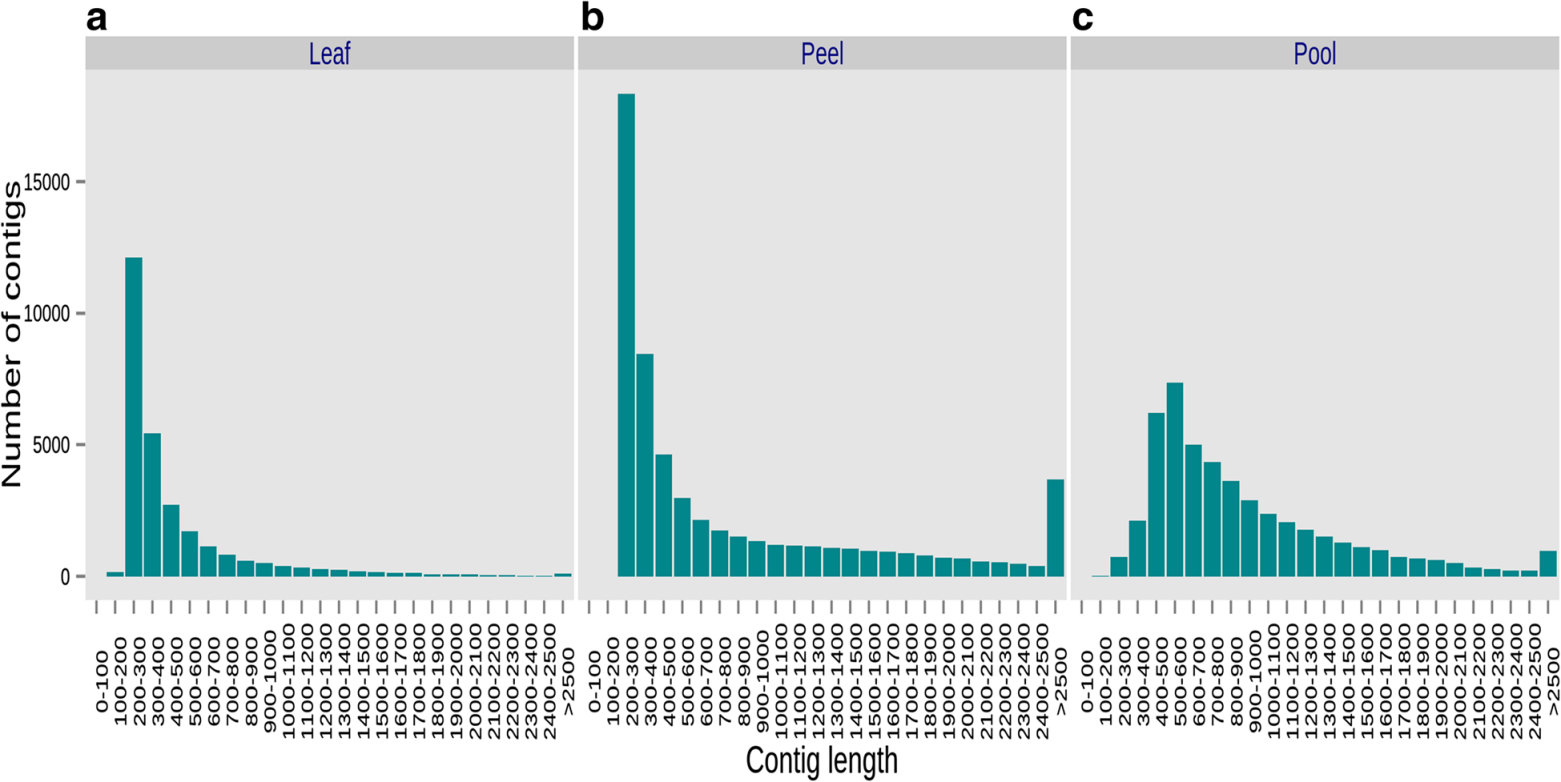
Distribution of contig lengths and comparison with two published mango transcriptomes. The distribution of contig lengths from three assemblies was plotted: Leaf (**a**), Peel (**b**), and Pooled (**c**) of tissues. The distribution of consensus contig lengths is drawn as 100-bp long bins

The sequencing of ‘Keitt’ using 454-GS FLX Titanium was yielded 1,329,313 reads. After filtering out low quality and empty reads, *de novo* assembly was performed on 1,113,875 reads resulting in 60,997 contigs. These contigs were then reassembled into super-contigs using the CAP3 program [34]. Ten percent of the contigs (6396) were assembled into super-contigs most (90 %) of which comprised 2 to 3 contigs. Altogether, the assembled ‘Keitt’ transcriptome contained 47,956 singleton contigs and super-contigs (hereafter mango contigs). We compared the results of the assembly in this work with two additional published assemblies that were based on a different sequencing strategy [21, 23]. Those transcriptomes were sequenced from RNA samples of leaf (hereafter Leaf transcriptome) [21] and fruit peel (hereafter Peel transcriptome) [23] tissues using Illumina technology followed by *de novo* assembly of short reads. Ninety percent of the contigs were 412, 219, and 223 bp or longer and half were at least 757, 321, 438 bp long for Pool, Leaf and Peel transcriptome assemblies respectively (Fig. 1). Both statistics suggested that the contigs of the Pool transcriptome are twice as long as those of the Leaf and Peel transcriptome assemblies [21, 23]. Obviously, the novel Pool transcriptome in this study significantly contributes to the length of available transcripts.

**Fig. 2:**
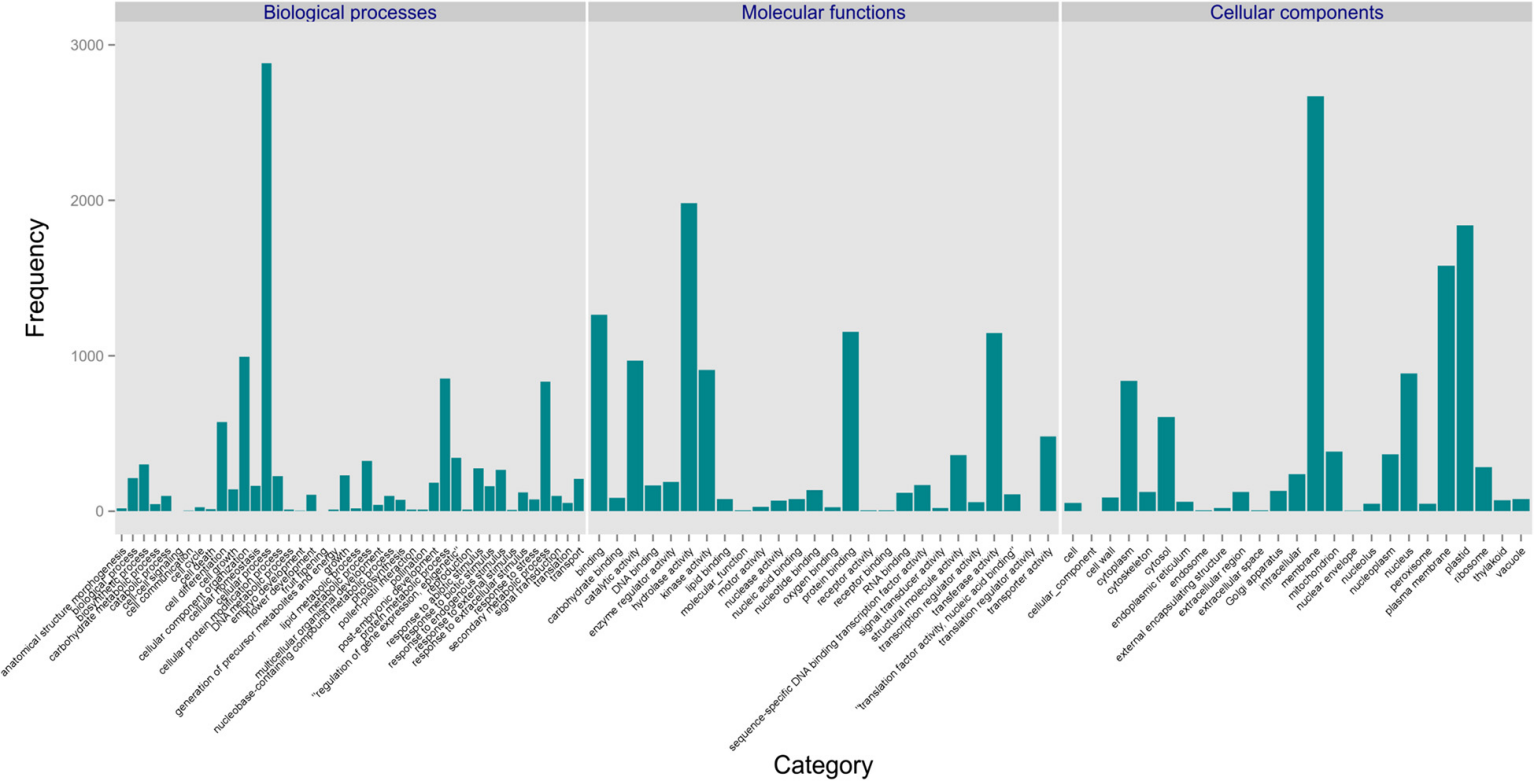
Comparison of mango gene ontology categories in three transcriptome assemblies. Contigs were annotated by running blast search against ‘nr’ database and then performing mapping to Slim-GO categories by Blast2GO. The distribution of contigs of the three ontologies, biological processes, molecular functions, and cellular components was plotted for transcripts that were included exclusively in the transcriptome from the pool (Pool only) of tissues (root, leaf, flower and fruit developmental stage 3; turquoise bars)

Functional annotation was also performed. First, the functional annotation of the Pool transcriptome resulted in a successful list of 40,971 hits (85 %) as a result of similarity searches against ‘Gene Bank’, ‘TAIR’, and ‘UniProt’ protein databases (Table 1). Second, by comparison to Leaf and Peel transcriptomes, we could investigate what are the common functionalities between leaves and fruit peel and assess whether novel transcriptome information was revealed in the Pool transcriptome. A reciprocal blast was run between the Pool and Peel transcriptomes, and between Pool and Leaf transcriptomes. The best hits were taken as the homologous transcripts. The number of Pool transcripts that were homologous to the Peel transcripts only was 10,251 whereas 3860 Pool transcripts were homologous to Leaf transcripts only. The common subset of transcripts, i.e., the intersection of the Peel, Leaf, and Pool transcriptomes, included 8660 transcripts (Additional file 1: Table S1). Half of the transcripts in the Pool transcriptome (21,880; 49 %) had no homolog in either the Peel or Leaf transcriptomes. The excess of transcripts in the Pool transcriptome relative to the Peel and Leaf transcriptomes could reveal new functionalities. Therefore a comparison of gene ontology (GO) functional categories between the common subset of transcripts and the rest of the transcripts might reveal whether or not new functionalities have been rendered. Figure 2 illustrates the distribution of GO-slim categories in the Pool transcriptome. In general, most of the GO-slim categories existed in both subsets of the Pool transcriptome. However, three transcripts related to cell communication category in the biological process ontology appeared exclusively in the Pool transcriptome as were five other transcripts related to the extracellular space.

**Table 1:** Number of mango contig homologous hits

	Non -redundant GeneBank proteins (nr)	TAIR	UniProt	Union of three database hits
Pool	40,795	34,918	30,684	40,971
Pool and peel intersection	17,366	16,079	13,173	17,423
Pool and leaf intersection	12,022	11,351	9390	12,038
Pool, peel and leaf intersection	8371	8074	6669	8380

### Transcriptome variation

**Fig. 3:**
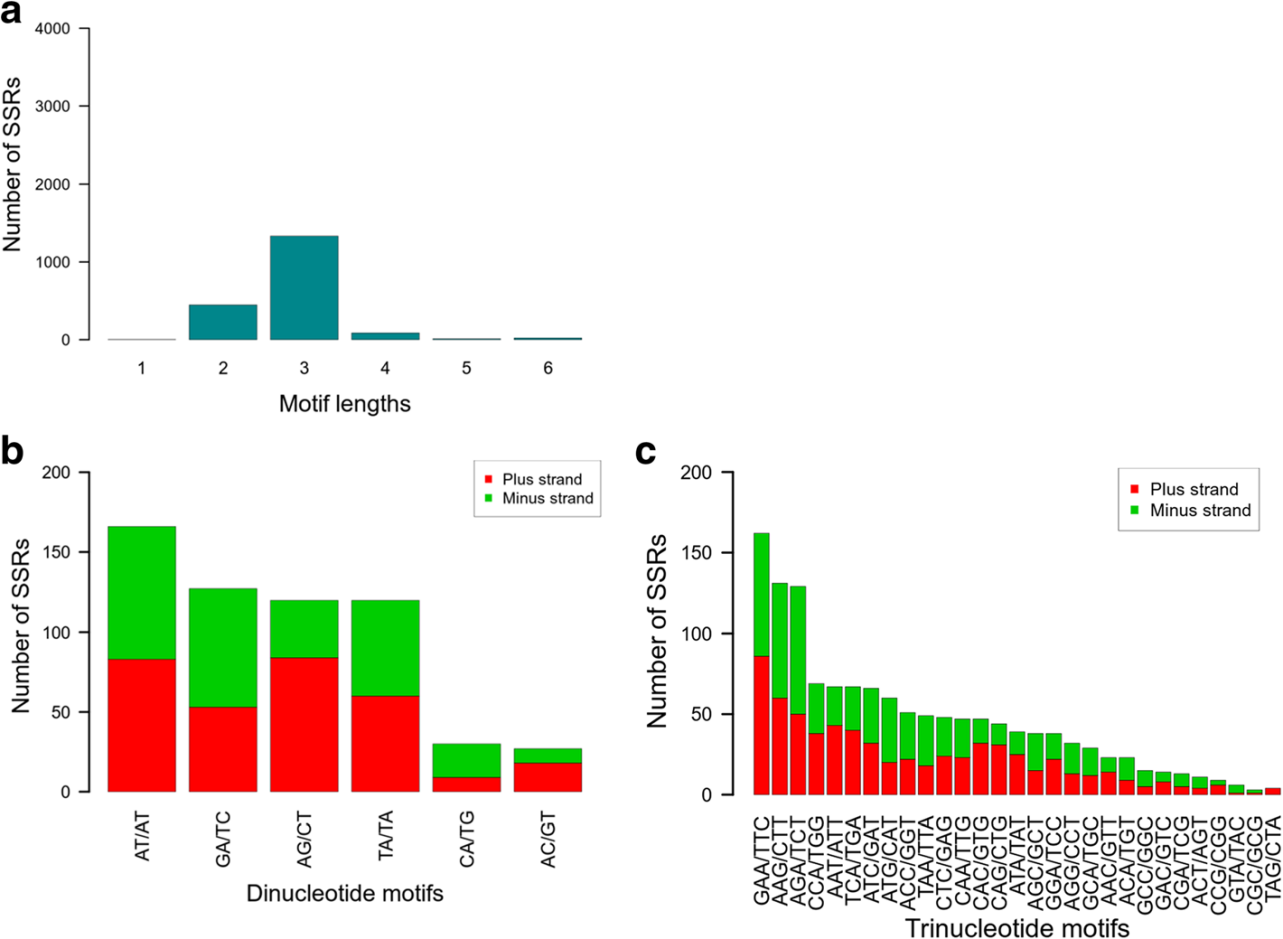
SSR length and motif distribution. The number of mono- to hexanucleotide SSR motifs was counted (**a**). The nucleotide compositions of the most frequent motifs (di- and trinucleotide motifs) were determined for each type and are illustrated in a bar plot for dinucleotide (**b**) and trinucleotide motifs (**c**). Motifs that are reverse-complementary were plotted as stacked bars: “plus strand” (*red*) and “minus strand” (*green*)

SSRs and single-nucleotide polymorphisms (SNPs) are highly useful in plant genetics and breeding for the construction of linkage maps and MAS [35, 36]. Therefore, we focused on the repertoire of SSRs and SNPs in the mango transcriptome. The number of SSRs found in the transcriptome was 1903. The SSRs were discovered in 4 % (1787) of all transcripts of the Pool transcriptome (Additional file 2: Table S2). The lengths of the SSR motifs ranged from 1 to 6. Most of the SSRs are trinucleotides (70.1 %) followed by dinucleotide (23.5 %) (Fig. 3a). The most frequent dinucleotide motif was (AT/AT)n with a frequency of 166 out of 590 followed by (GA/TC)n, (AG/CT)n, and (TA/TA)n (Fig. 3b). The least frequent motifs (only 10 %) were (CA/TG)n and (AC/GT)n. The three most frequent trinucleotide motifs are (GAA/TTC)n, (AAG/CTT)n, and (AGA/TCT)n with the proportions of 12, 10 and 10 % of all trinucleotide motifs, respectively (Fig. 3c). The novel SSRs, in this study, are expected to greatly enrich the mango community reservoir of SSRs that have already been reported [8, 9, 13, 14, 18, 20, 37, 38]. The SSRs in those studies were used as a genetic tool to investigate diversity in local germplasm collections. In general, those studies were based on a few SSRs and the frequency of the SSR motifs in the genome was not reported. Thus the SSR motifs could not be compared. However, the pattern of SSR motifs is known to be species-specific [39, 40]. Thus, a discovery of the same pattern of SSR motifs in the same species can strengthen the SSRs’ reliability. Previously reported SSR motifs were congruent with the motifs that were reported here verifying their reliability. For example, a study of Australian collection’s diversity identified 100 SSRs within approximately 24K expressed sequence tags (ESTs) [20]. The trinucleotide motifs were more frequent than dinucleotide motifs in both the Australian collection in the present study. Moreover, the motif patterns that were reported as the preponderant ones were congruent with our observations. The trinucleotide motif, (AAG/CTT)n, was ranked as the most and second most frequent in the Australian study and in our study, respectively, and the dinucleotide motif, (AG/CT)n, was ranked as the most and third most frequent, respectively. The list of SSRs discovered might be useful for MAS and genetic surveys. However, in spite of the fact that NGS can be used for SSR discovery, high-throughput technologies (microarrays and NGS) are more available for SNPs [26, 35, 41]. Therefore, in terms of parallel genotyping the available technologies tilt the balance in favor of using SNPs as markers rather than SSRs.

In the recent years, with the evolution of next generation sequencing, many studies have developed SNP markers for marker-assisted breeding [32, 42–45]. NGS has leveraged the genome-wide SNP discovery in non-model organisms such as spruce [46], apple [47, 48], and pomegranate [31]. However, no study of SNP development for mango has been reported yet. In the present work, two mango accessions’ transcriptomes (‘Keitt’, ‘Tommy Atkins’) were resequenced and aligned to a *de-novo* assembled transcriptome as a reference. The analysis resulted in the discovery of 332,016 SNPs (Additional file 3: Table S3) using VarScan [49]. The polymorphism type of those loci for the two accessions’ transcriptomes can be either polymorphic, i.e., heterozygous (He) or non-polymorphic, i.e., homozygous (Ho). The possible combinations of the genotype calls for the two transcriptomes fall into four categories: both transcriptomes are homozygous (HoHo), ‘Keitt’ is heterozygous and ‘Tommy Atkins’ is homozygous (HeHo), ‘Keitt’ is homozygous and ‘Tommy Atkins’ is heterozygous (HoHe), and both transcriptomes are heterozygous (HeHe). Note that if both transcriptomes are homozygous, they are homozygous for different alleles. The distribution of SNPs into these categories was 24,136, 33,554, 164,454, and 109,872, respectively. Thus ‘Tommy Atkins’ is more polymorphic than ‘Keitt’. As expected more SNPs were discovered in the flanking regions of the open reading frames (ORFs; hereafter outORF), than within them (hereafter inORF). The ratio of outORF to inORF SNPs was 2.18 on the average. This ratio was uniformly maintained in all SNP categories except in the HoHo category where the ratio of outORF to inORF SNPs is two and it was found to be significantly smaller (*χ*^2^ test; df =3; *p*-value <0.001) than 2.18 as a result of a slight increase of inORF SNPs. Herein we described the first set of SNP markers for mango. The closest fruit tree relative of mango with a published genome, *Citrus sinensis*, is as polymorphic as mango [50]. The genome project of the sweet orange reported 1.06 million SNPs in the entire genome with one-third are in genic regions [51]. Like orange, 70 % of the transcripts included at least one SNP while only 63 % included at least one SNP in the exonic regions. Other studies of fruit trees reported much less polymorphism in expressed regions: 6500, 71,482 and 23,742 in pomegranate [31], apple [52], and eucalyptus [27], respectively. These findings confirm previous results that mango is a highly heterozygous (or polymorphic) species [7, 12, 53].

### Germplasm kinship

An overwhelming number of SNPs derived from the genic region of the genome may be useful in the future for genome-wide association studies (GWAS). However as a preliminary step to such studies, a survey of the structure and diversity of the mango collection is required [54]. A subset of 239 high quality SNPs was used for genotyping 74 accessions of the Israeli mango collection, one SNP per contig. The SNPs subset was not biased toward “inORF” or “outORF” types of SNPs (*χ*^2^-test; df = 1; *p*-value = 0.74) and was therefore representative. As reported in previous studies of collections, mangoes are highly polymorphic [13, 19]. The median polymorphism information content (PIC) was 0.4 whereas less than 1 % of the applicable SNPs were of minor allele frequency (MAF) value <0.05. Thus most of the SNPs were polymorphic in the Israeli mango collection although they were discovered in only two accessions, ‘Keitt’ and ‘Tommy Atkins’. That is reasonable presuming that mango is highly polymorphic.

**Fig. 4:**
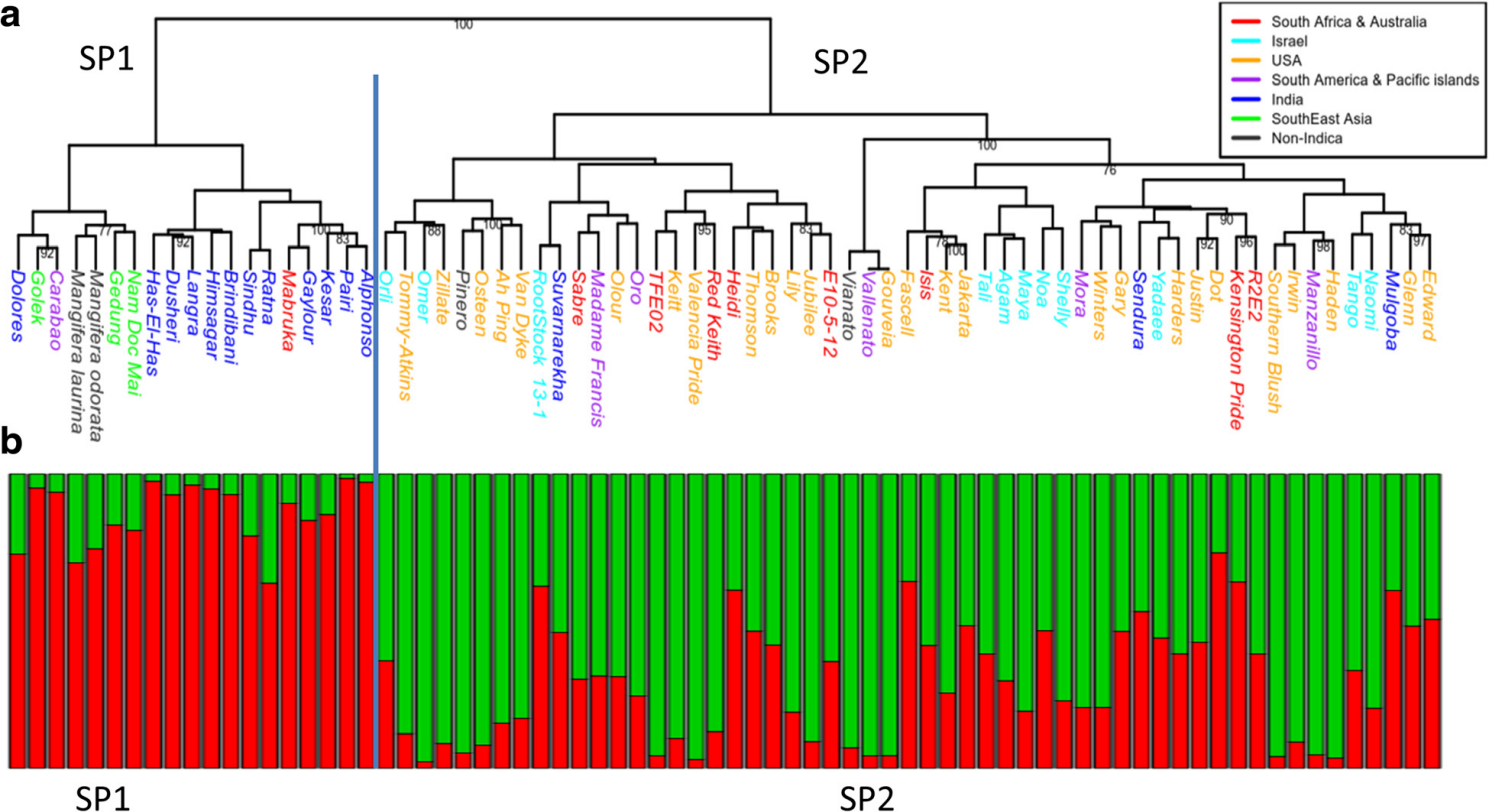
Dendrogram and genetic structure of 74 accessions in the Israeli mango germplasm collection. Genotyping of 74 mango accessions from the Israeli mango collection was performed with 239 SNPs. The genotyping results were used to classify the accessions into sub-populations and reveal their genetic structure. **a** Classification was performed by drawing a dendrogram based on 1- proportion of shared alleles (PSA) as a genetic distance. Only confident branches with bootstrap values ≥90 were assigned. The two major groups are notated as subpopulation 1 (SP1) and as subpopulation 2 (SP2). **b** Genetic structure was revealed by STRUCTURE program with K = 2 as found by simulation and ΔK likelihood method. The division of STRUCTURE’s Q-value bar plot into two (*vertical blue line*) corresponds to the two major significant clusters in the dendrogram. Note that the Y-axis (not plotted) scale is between 0 and 1 and represents proportion

The Israeli germplasm collection comprises cultivars that were originated from India, Southeast Asia, South America and the Pacific islands, Florida, Australia, and from elite local hybrids. A dendrogram based on the proportion of shared alleles distance classified the accessions in the mango collection into two genetic subgroups. The dendrogram (Fig. 4a) split the mango collection into two major clusters: 1) a small one that comprises most of the Indian accessions clustered together with accessions from Southeast Asia (SP1), 2) and a larger one which comprised of the Floridian, South African, Australian, local (Israeli) and South American accessions (SP2). This division separates Indian and Southeast Asian accessions from the rest. In other words, the mango accessions that are cultivated in the western part of the world can be genetically separated from those that are cultivated in its eastern part of the world. Due to the fact that the origin of mango has been suggested to be from the eastern part of the world [55], SP1 might be more related to the landrace mangoes. Three accessions from India fell within the Floridian-Israeli (SP2) cluster. ‘Mulgoba’ was reported as the parent of the Floridian cultivar ‘Haden’ and as a putative parent of other Floridian variants [3]. Moreover, ‘Haden’ has been suggested to be the parent of many other Floridian accessions [3]. Thus, ‘Mulgoba’ is the ancestor of most Floridian accessions. Recently a new study was published and reported about 387 mango accessions from all over India. In that study, the cultivar ‘Suvarnarekha’ was reported from South India as was ‘Mulgoba’ and they both were clustered together in a dendrogram by their geographical origin [14]. To the best of our knowledge no record exists of the origin and the genetic similarity of the third Indian accession, ‘Sendura’. Moreover, the number of subpopulations estimated by Evanno’s method [56] was K = 2. Most of the accessions from India which were clustered together were genetically homogeneous (Fig. 4b; mostly red bars), while the three accessions from India that were included in the cluster with the Floridian and Israeli accessions are highly admixed (Fig. 4b; red/green bars). Ravishankar et al. [14] showed that the Indian collection can be divided into two subpopulations corresponding to the geographical classification of south/west and north/east. Moreover, the south/west can be further divided into two sub-populations. It is not clear whether the additional genetic division is correlated with south and west geographical regions. However assuming this correlation would explain the fact that in the SP1 cluster, the Indian accessions were from north, east, and two from west, while the Indian accessions from the south were included separately in the SP2 cluster (Fig. 4a).

In contrast to the mango accessions’ origin, there was no clear division observed between poly- and monoembryonic accessions in the SP1 and SP2 clusters. SP1 comprised 13 and five mono- and polyembryonic accessions respectively (one was undefined). SP2 comprised 40 and 11 mono- and polyembryonic accessions respectively (four were undefined). No significant difference (Fisher’s exact test; *p*-value = 0.47) was observed between the proportions of poly- and monoembryonic accessions in the two clusters.

### Mango diversity

The two major clusters in the dendrogram were compared for their genetic diversity. The expected heterozygosity of SP1 (median = 0.28) is significantly smaller (Wilcoxon test, *p*-value <0.001) than the expected heterozygosity of SP2 (median = 0.43). The accessions in SP1 are in Hardy-Weinberg equilibrium (HWE) with a median F_S_ of -0.05 (Wilcoxon test, p-value = 0.13). In contrast, slight outbreeding was estimated for the accessions in SP2 with a median of F_S_ = -0.1 (Wilcoxon-test; *p*-value <0.001). Both F_S_ values were close to zero and slightly negative, suggesting that mango accessions in these clusters are not prone to inbreeding. The SP1 cluster that was enriched in accessions from Southeast Asia and India, i.e., suggested mango’s origin [55], and its accessions had probably been under cultivation longer duration than the accessions in SP2. Therefore the result that they were in HWE is acceptable. In contrast the SP2 cluster deviated from the HWE. One explanation is that as a group, the accessions in the cluster as a group appeared to be under shorter duration of cultivation. Alternatively, one might suggest that SP1 is comprised of accessions that are more related to landraces (note that non-indica mangoes are included). The SP2 cluster comprises of accessions that were subjected to breeding efforts. This may be one of the reasons that SP1 is under HWE while SP2 deviates from it. A supportive evidence that SP2 is a young subpopulation lies in the estimation of a F_ST_ value that is only slightly greater than zero (median = 0.03; Wilcoxon-test; *p*-value <0.001) which suggests that SP2 is only in the beginning of its differentiation. Small F_ST_ values, such as the one shown in this study, were previously suggested by three other studies [11, 18, 57] of the Indian and Colombian mango collections using SSR and RAPD markers respectively. The SP2 cluster is also more diverse than the SP1 cluster. The genetic structure analysis (Fig. 4b) illustrated that accessions in the SP1 cluster have come from a narrow genetic background whereas the Indian-originated accessions in the SP2 cluster are more admixed. An optional explanation for this might relate to the possibility that the founder of the SP2 subpopulation (‘Mulgoba’) was probably a hybrid of the two subpopulations described in Ravishankar and colleagues’ study [14] and therefore heterozygous.

Finally, two non-indica species of the genus *Mangifera* were included in this study (*Mangifera laurina* and *Mangifera odorata*); they clustered together with SP1 subpopulation that contained mainly accessions that are cultivated in Southeast Asia and India. This supports the claim that the Southeast Asia and India is the origin of *Mangifera indica* [55] and that the accessions in SP1 are closer to landraces than the ones in SP2.

## Conclusions

We have established a sequence for the mango transcriptome from a pool of tissues. This transcriptome was not reconstructed to study expression but rather served as a reduction in complexity for variation discovery. It was used as a reference to align resequencing of two commercially important mango accessions, ‘Keitt’ and ‘Tommy Atkins’, constituting a resource for genetic variation discovery. The annotation and the SSR motifs were congruent with the existing knowledge in the literature. The discovered SNPs established a large pool genetic variation in mango. A subset of this pool was shown to be applicable for studying diversity in the Israeli mango collection and for dividing it into two subpopulations, i.e., two genetic groups. The SP1 cluster comprised a Southeast Asian and Indian accessions and was suggested to arise from a narrow genetic background. Yet it was found to be in HWE, probably due to a long duration of cultivation. In contrast the SP2 cluster comprised mainly accessions cultivated in the western world except for three Indian accessions, one of which had been reported to be the ancestor of many Floridian mangoes. The structure analysis based on the SNP markers suggested that the three Indian mango accessions are an admixture. Consequently, most of the descendent cultivars are admixtures as well. In contrast to SP1 accessions, those in SP2 were not in HWE. We suggest that the different results are probably due to difference in duration of cultivation, although this was not strongly supported by the results. We believe that the novel set of SNPs is valuable for mango because that they have been polymorphic in the Israeli mango collection and they enabled us to recapitulate the mango’s diversity.

## Methods

### Plant material

Mango accessions from the Israeli mango germplasm collection were used in this study. The collection is comprised of accessions from different regions of the world as well as promising lines identified through the Israeli breeding program. A list of the accessions that were included in this study is provided in (Additional file 4: Table S4). All accessions were 15–20 years old, grafted on the 13/1 rootstock. Trees were grown in sandy soil at the Volcani Experimental Orchard in Volcani Center, Israel. All samples were collected, immediately frozen in liquid nitrogen and stored at −80 °C until use.

### RNA isolation

RNA was purified from several tissues of ‘Tommy Atkins’ and ‘Keitt’ trees (young leaves, young inflorescences, fruitlets, flesh and peel of mature fruit). Total RNA was isolated using a hexadecyltrimethyl ammonium bromide (CTAB)-based method [58]. Tissue (2–3 g) was ground in liquid nitrogen and extracted in 20 ml extraction buffer (0.1 M Tris, 25 mM EDTA, 2 % (w/v) CTAB, 0.2 % polyvinylpyrrolidone [PVP], 2 M NaCl, 0.2 % ß-mercaptoethanol, pH 8.0) pre-warmed to 65 °C. After two phenol:chloroform extractions, RNA was precipitated with 2.5 M LiCl, and re-suspended with 1 ml SSTE (0.5 % SDS, 1 M NaCl, 10 mM Tris, pH 8, 1 mM EDTA, pH 8). RNA was re-extracted twice with phenol:chloroform and precipitated in 70 % ethanol. Purified RNA was treated with RQ1 RNase-free DNase I (Promega) according to the manufacturer’s instructions, followed by another extraction and precipitation. The RNA was assessed for integrity and quantified on a NanoDrop spectrometer and by separation on a 1.2 % agarose gel.

### Isolation of genomic DNA

Genomic DNA was isolated from young mango leaves (2 g) ground in liquid nitrogen and extracted with 15 ml of extraction buffer (100 M Tris, pH 8.0, 1.5 M NaCL, 3 % CTAB, PVP, 1 % ß-mercaptoethanol) and 15 ml of chloroform: isoamyl alcohol. Following a second extraction with chloroform: isoamyl alcohol, DNA was ethanol-precipitated, treated with 20 units of ribonuclease A (Sigma), precipitated and resuspended in water. The DNA was quantified in a NanoDrop spectrometer and by separation on a 0.8 % agarose gel.

### High throughput sequencing

‘Keitt’ total RNA samples from the different tissues were mixed evenly and ran on one plate of the 454-Titanium platform. Construction of two cDNA libraries and 454 pyrosequencing were carried out at the W.M. Keck Center for Comparative and Functional Genomics, Roy J. Carver Biotechnology Center, University of Illinois at Urbana-Champaign. Briefly, mRNA was isolated from 20 μg of total RNA with the Oligotex kit (Qiagen, Valencia, CA). The mRNA-enriched fraction was converted to a primary cDNA library with adaptors compatible with the 454 system as previously published [59]. After library construction, the library was quantified using a Qubit fluorimeter (Invitrogen, CA) and average fragment sizes were determined by analyzing 1 μl of the samples on a Bioanalyzer (Agilent, CA) using a DNA 7500 chip. The libraries were diluted to 1 × 10^6^ molecules/μl and pooled. Emulsion-based clonal amplification and sequencing on a full plate of the ‘454 Genome Sequencer FLX+’ system were performed according to the manufacturer’s instructions (454 Life Sciences, Branford, CT). Signal processing and base calling were performed using the bundled 454 Data Analysis Software v2.6. The read outcome was used to create a mango transcriptome as a reference for alignment of resequenced ‘Keitt’ and ‘Tommy Atkins’ total RNA mixture isolated from several tissues in equal amounts. Those RNA samples were prepared with Illumina’s ‘TruSeq RNAseq Sample Prep kit’, quantified by qPCR, and sequenced for 100 cycles on a HiSeq 2000 using a ‘TruSeq’ SBS sequencing kit version 3. To get a lane-independent yields, ‘Keitt’ and ‘Tommy Atkins’ RNA samples were initially tagged and then mixed evenly and were run on two separate lanes. The sequence reads from those lanes were used for discovery of genetic variation.

### De novo transcriptome assembly and functional annotation

Raw sequence reads of the 454-FLX GS Titanium platform were pre-processed by “SFF_extract” (http://bioinf.comav.upv.es/sff_extract/) and arguments for removing the adaptors and clipping the poly-A were applied. Reads were assembled by a stable version of MIRA, v3.2 [60]. For the MIRA run, we used the “Do-What-I-Mean” (DWIM) set of parameters as follows: “denovo, est, normal, 454”, ‘assume_snp_instead_repeat’, ‘clip_polyat’ and ‘force_nonIUPACconsensus_perseqtype’ options on, and ‘min_reads_per_group’ = 8, ‘min_neighbour_qual’ = 25 and ‘min_groupqual_for_rmb_tagging’ = 30. One of MIRA features involves splitting mRNA unigenes into splice variants especially for polymorphic variants. Therefore a second assembly run on MIRA’s contigs was performed by CAP3 [34] to produce super-contigs. Both super-contigs and the singletons, which are the MIRA’s contigs were designated reference contigs. Contigs were deposited in the transcriptome shotgun assembly (TSA) sequence database [TSA: SAMN02905156, SAMN02947194].

All contigs were searched for open reading frames (ORFs) by the “getorf” program of the EMBOSS package [61]. The longest ORF with start and stop codons was chosen for each contig with a minimum cutoff of 67 amino acids.

A sequence-similarity search of contigs was run against the non-redundant (nr) protein database using blastx with a filter of e-value <10^−5^. Best hits were further mapped to GO-slim by Blast2GO [62] and only hits with Blast2GO annotation score >55 were scored (Additional file 2: Table S2). Mapping of the mango peel transcripts [23] to the transcripts of the pooled tissues in this study was performed by blast search for all transcripts of the peel against pool and vice versa, and selecting the reciprocal best hits. Similar but separate mapping was performed with the transcripts of mango leaves [21].

### SNP and SSR discovery

Read results from ‘Keitt’ and ‘Tommy Atkins’ mRNA resequencing using Illumina HiSeq 2000 were mapped to the ‘Keitt’ reference-transcriptome contigs using bowtie2 (http://bowtie-bio.sourceforge.net/bowtie2/index.shtml). SNPs were discovered using Varscan [49].

SSR scanning was performed on the 47,956 reference contigs. MIcroSAtellite (MISA) identification tools (http://pgrc.ipk-gatersleben.de/misa/) and SciRoKo [63] were run with default parameters.

### Genotyping assays

A subset of 472 SNPs was chosen for further analysis by maximizing sequence coverage of 1 SNP per contig. SNP assays for all 472 SNPs were developed by Fluidigm based on the genetic variation that was found between ‘Keitt’ and ‘Tommy Atkins’. The assays were run according to the manufacture’s instructions on an EP1 platform using ‘96 × 96’ chips following standard Fluidigm protocols (http://www.fluidigm.com) with a minor modification of four no-template control (NTC) samples instead of one. The SNP assays were used to screen the 74 accessions’ DNA samples by running on ‘FR96.96’ arrays of the EP1 Fluidigm platform according to the manufacturer’s instructions (http://www.fluidigm.com).

### Data analysis of mango diversity

To exclude bad samples and failed marker assays, samples that had more than 10 % “No Call” and assays with more than 30 % “No Call” were removed. The remaining subset was submitted for the downstream analysis. The PIC was calculated as [64].

$$ \mathrm{PIC}=1-{\displaystyle \sum {\mathrm{p}}_{\mathrm{i}}^2} $$

where i is the i-th allele.

### Germplasm accession classification and diversity

To assess the relationship between different mango accessions, we estimated the genetic distance as D = 1-proportion of shared alleles (PSA). PSA was calculated as

$$ PSA=\frac{{\displaystyle {\sum}_{i=1}^L}P{S}_i}{2*L} $$

where PS is the proportion of shared alleles for each locus and L is the total number of loci [65].

Hierarchical clustering was performed on a pairwise D distance matrix and the “ward” agglomerative method [66] was applied. The confidence limits of the tree topology were calculated by applying bootstrap method (1000 resampling of loci). To count the number of bipartitions fitting the tree we used the “ape” R-package [67] and presented the bootstrap values as percentages.

The subpopulation structure underlying the germplasm collection was estimated by running a simulation of STRUCTURE software v2.3.3 [68] with 5000 burn-in periods and 50,000 repetitions. The number of populations, K, was inferred by running the simulation of K = 1 to K = 10 (20 runs for each K) and using the likelihood method of ΔK [56].

The fixation indices F_S_ and F_ST_ [69] were calculated as

$$ Fs=\frac{H_{exp}-{H}_{obs}}{H_{exp}} $$

where F_S_ is the fixation index of each subpopulation, H_obs_ is the observed heterozygous types and H_exp_ is the estimated heteozygosity under HWE,

$$ {F}_{ST}=\frac{H_S-{H}_T}{H_T} $$

where F_ST_ is the genetic differentiation of a subpopulation due to genetic drift, H_S_ is the weighted average of all subpopulations’ expected heterozygozity, and H_T_ is the expected heterozygosity in the entire population (germplasm collection).

## Availability of supporting data

The dataset supporting the results of this article is available in the NCBI TSA (Transcriptome Shotgun Assembly Sequence Database, http://www.ncbi.nlm.nih.gov/genbank/tsa) repository under the accession numbers of BioProject: PRJNA254771, BioSample: SAMN02947194, and BioSample: SAMN02905156. These data can be found under a search in the Nucleotide repository at the NCBI site.

## Additional files

Additional file 1: Table S1. Mango transcriptome annotation. (XLSX 16205 kb) Additional file 2: Table S2. Mango simple sequence repeats. (XLSX 102 kb) Additional file 3: Table S3. Mango SNP list. (XLSX 14669 kb) Additional file 4: Table S4. List of accessions in the Israeli germplasm collection and metadata. (XLSX 11 kb)

## Abbreviations

MAS: marker-assisted selection
NGS: next generation sequencing
ORF: open reading frame
PIC: polymorphism information content
PSA: proportion of shared alleles
